# Clinical, Radiological, and Lung Function Characteristics of Post-tuberculosis Bronchiectasis: An Experience From a Tertiary Care Center in India

**DOI:** 10.7759/cureus.34747

**Published:** 2023-02-07

**Authors:** Jyoti Bajpai, Surya Kant, Ajay Verma, Darshan K Bajaj

**Affiliations:** 1 Respiratory Medicine, King George's Medical University, Lucknow, IND; 2 Respiratory Medicine and Pulmonary Critical Care, King George's Medical University, Lucknow, IND

**Keywords:** ct thorax, cough, tuberculosis, inflammation, non-cystic fibrosis bronchiectasis

## Abstract

Introduction

Among chronic respiratory diseases, bronchiectasis is one of the important causes of mortality and morbidity in developing countries.

Objective

This study aimed to assess the clinical, radiological, microbiological, and pulmonary function profiles of adult patients with post-tubercular bronchiectasis.

Methods

We enrolled 138 patients with bronchiectasis confirmed by high-resolution CT scans from July 2017 to August 2018.

Results

A total of 138 patients with bronchiectasis were enrolled. The data from 132 patients were analyzed; six patients were excluded from the study. The mean age of post-TB bronchiectasis (post-tuberculosis bronchiectasis) patients was 36.08±13.08, which was lower than the non-tuberculosis bronchiectasis group. The proportion of the male population was more in the post-TB bronchiectasis group (54.55% vs. 37.88%, p=0.48). Smoking prevalence was high in post-TB bronchiectasis (27.27% vs. 12.12%, p=0.04). The predominant symptom was cough in the post-tubercular bronchiectasis group (48.5% vs. 41.7%, p=0.019). The history of the recurrent common cold was seen most frequently in non-post-tubercular bronchiectasis (40.9% vs. 12.9%, p=0.001). The most common radiological variant of bronchiectasis found in all patients was a cystic type (75%). The most common site of involvement was the left lower lobe, followed by the lingula in all patients and post-tuberculosis bronchiectasis patients. Pulmonary function on spirometry revealed obstructive, restrictive, and mixed patterns in 55%, 25%, and 15%, respectively. Patients with post-tuberculosis bronchiectasis had lower lung function post-FEV1/FVC (forced expiratory volume in one second/forced vital capacity) ratio (70.31±15.56 vs. 76.85±11.82, p=0.015). Binary multivariate logistic regression analysis showed that only recurrent cough cold was a significant independent risk factor for post-TB bronchiectasis.

Conclusion

Post-tuberculosis, and bronchiectasis followed by post-infectious causes, were the most common causes of bronchiectasis and poor lung function.

## Introduction

Bronchiectasis is a chronic respiratory disease and an emerging global health problem. It is an umbrella term defined by abnormal, permanent dilatation of bronchi seen on the CT thorax characterized by respiratory symptoms, cough, sputum production resulting from chronic infection, and inflammation [1]. The trinity of bronchiectasis is an infection, airway inflammation, and destruction. It is a heterogeneous disease with different etiologies associated with various syndromes. Due to the heterogeneous nature of the illness and varied etiology, there are differences in the presentation and natural course of the disease. The growing prevalence of bronchiectasis in recent years is an area of concern. In Europe and North America, the prevalence of bronchiectasis has been shown to range from 53 to 566 per 100,000 people [2,3]. In Europe and the USA, the disease prevalence has increased by more than 40% in the last ten years [4].

Even though there has been significant improvement in our understanding of this condition, most of the large-scale epidemiological databases on bronchiectasis have come from the USA, Australia, and European nations. Small cohorts from China and South America suggest that the characteristics of patients from low­ income and middle­ income countries might differ from those in Europe. There is no data to define the exact burden of bronchiectasis in India. The first multicentre, prospective, and observational European Multicentre Bronchiectasis Audit and Research Collaboration (EMBARC) India registry highlighted the etiology, disease severity, microbiology, treatment, and demographic characteristics of bronchiectasis patients [5]. Data from European countries found that 40% of bronchiectasis cases are idiopathic and are most commonly seen in female and older age groups [6]. The EMBARC India registry revealed that tuberculosis is the most common etiology for bronchiectasis [5]. Post-tuberculosis bronchiectasis is an emerging phenotype in Asian countries. Tuberculosis (TB) and bronchiectasis are interlinked, although bronchiectasis can occur during tuberculosis or be a sequela of TB. A systematic review evaluated the prevalence of 35-86% in post-tuberculosis bronchiectasis patients diagnosed with CT imaging [7]. Here we describe the clinical characteristics, spirometry, and radiology of post-tuberculosis bronchiectasis.

## Materials and methods

**Figure 1:**
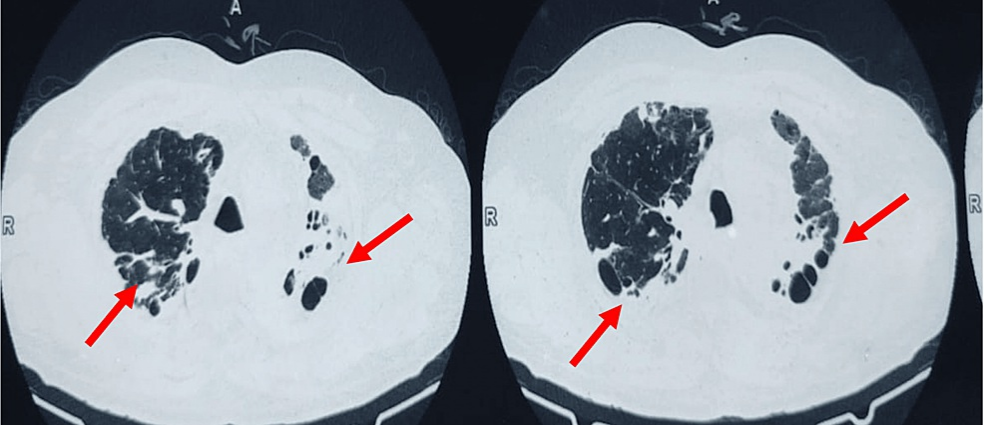
CT thorax shows bilateral bronchiectasis and fibro-cavitary (red arrow) changes in the upper zone with volume loss (post-tuberculosis)

This prospective, observational, non-interventional study enrolled 138 adult patients aged 18 years with bronchiectasis. Diagnosis of bronchiectasis was made on the clinical symptoms, cough, sputum production or recurrent respiratory infections, and CT thorax. Informed consent was taken from all enrolled patients. The patients with cystic fibrosis, bronchiectasis due to interstitial lung disease, hemodynamic instability, chronic kidney disease, acute myocardial infarction, and patients unable to consent were excluded from the study. The institution's ethics committee King George's Medical University approved the study. Post-TB bronchiectasis was assigned when history or clinical evidence of TB was evident and radiological findings of bronchiectasis were suggestive of TB, including scarring of the upper lobes, granuloma, and cavitation (Figure 1).

**Figure 2:**
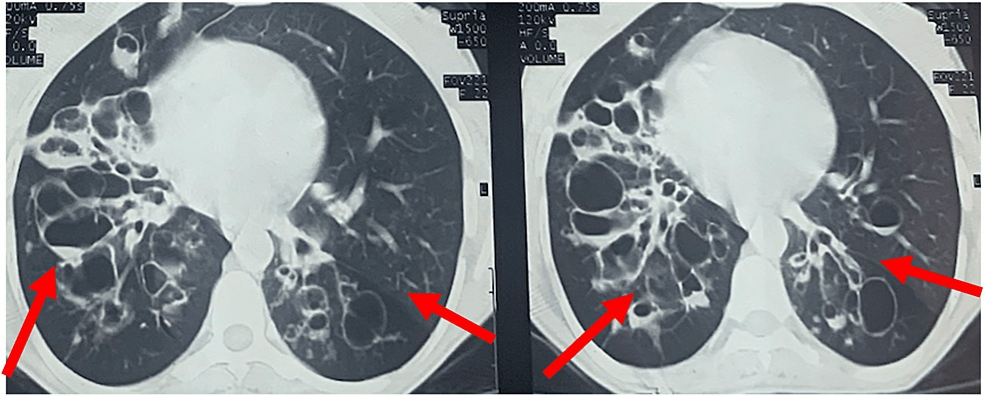
CT thorax of bilateral cystic bronchiectasis (red arrow) middle and lower zone

The post-infectious etiology was defined as bronchiectasis caused by a prior infection other than TB. (Figure 2).

According to the guidelines, idiopathic bronchiectasis was defined when no specific etiology was determined after a diagnostic workup. TB was ruled out in another bronchiectasis group by sputum smear examination for acid-fast bacilli (AFB) and molecular tests like cartridge-based nucleic acid amplification (CBNAAT). Line probe assay (LPA) tests were also conducted to rule out tuberculosis.

The demographic data, biochemical laboratory parameters, lung function testing, chest X-ray, HRCT thorax, and sputum microbiology were conducted. A pulmonary function test was done according to American Thoracic Society guidelines. The parameter percentage of predicted forced expiratory volume in one second (FEV1) was calculated using reference values for South Asian patients. Obstructive airway was defined by a FEV1:forced vital capacity (FVC) ratio of less than 0.7. Patients with a FEV1:FVC ratio of 0.7 or more and FEV1 and FVC less than 80% of the predicted values were classified as restricted lung disease [8]. A detailed history of respiratory symptoms was taken. Breathlessness was assessed on a modified Medical Research Council (mMRC) scale. The history and investigations exploring the etiology of bronchiectasis and allergic bronchopulmonary aspergillosis were diagnosed according to the guideline [9]. HRCT scans were done to diagnose bronchiectasis and other pulmonary abnormalities. 

Statistical analysis

The data were expressed as appropriate mean, standard deviation (SD), and number (%). All categorical data were compared by using the chi-square test. Continuous variables in the two groups were compared by t-test. The binary logistic regression was used to analyze the independent risk factor for post-TB bronchiectasis. The p-value <0.05 was considered significant. The statistical analysis was done using SPSS Statistics v. 21.0 for Windows (IBM Corp., Armonk, NY).

## Results

**Figure 3:**
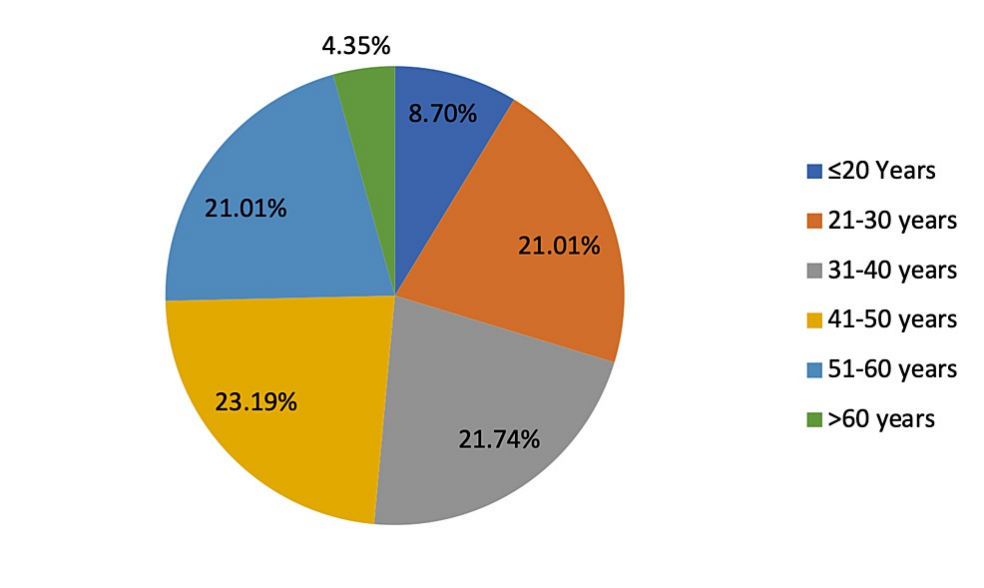
Distribution of patients according to different age groups

A total of 132 patients with bronchiectasis were enrolled in the study. The distributions of patients based on different age groups are shown in Figure 3.

**Figure 4:**
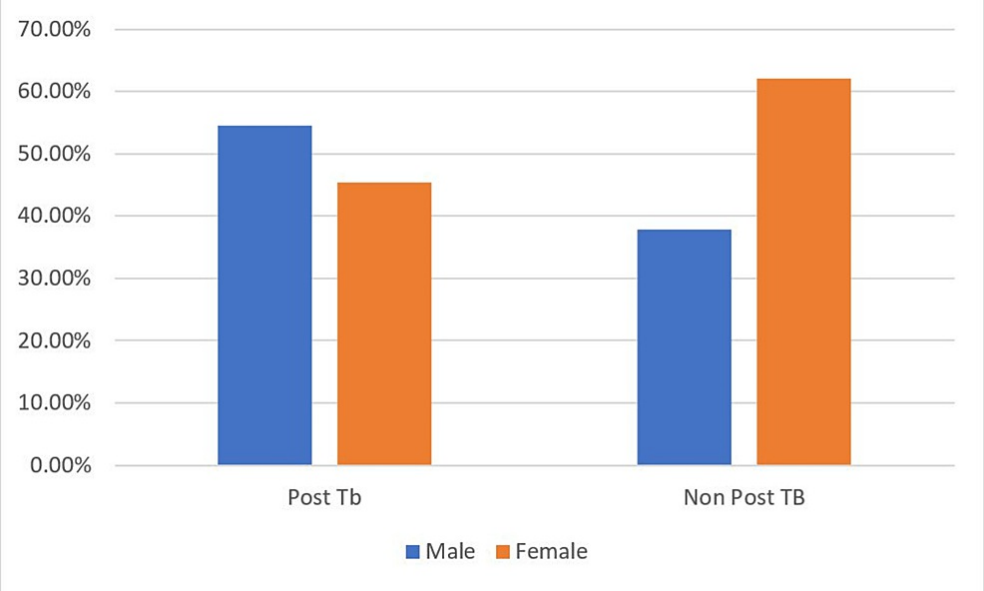
Sex distribution of post-tuberculosis (TB) bronchiectasis patients

The ages groups of ≤20 years, 21-30 years, 31-40 years, 41-50 years, 51-60 years, and >60 years had 8.70%, 21.01%, 21.74%, 23.19%, 21.01%, and 4.35% patients respectively. The majority of patients were between 20-60 years of age. The mean age of the participants was 48±20 years. A total of 58 (42.03%) patients were female, and 80 (57.97%) were male; the male:female ratio was 1:0.7 (Figure 4). 

**Figure 5:**
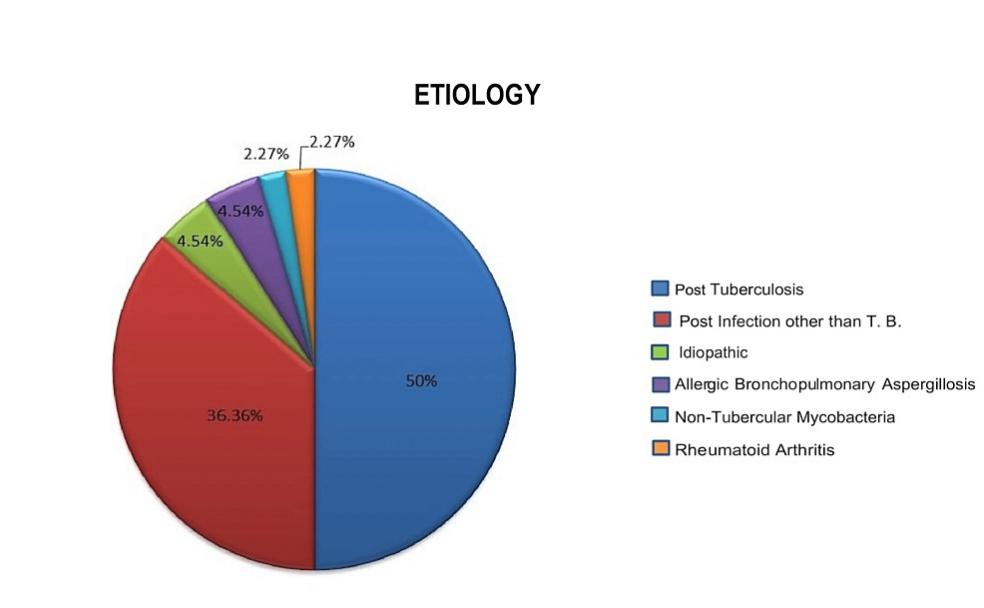
Different etiologies of bronchiectasis

Out of 132, a total of 66 (50%) patients were post-tuberculosis bronchiectasis group, and the rest comprise various aetiologies like post-infection other than tuberculosis (48; 36.36%), idiopathic (6; 4.54%), allergic bronchopulmonary aspergillosis (6; 4.54%), non-tubercular mycobacteria (3; 2.27%), rheumatoid arthritis (3; 2.27%). In our study group, immunodeficiency, alpha one antitrypsin deficiency, and other congenital disorders like tracheobronchomegaly were absent (Figure 5).

The patients were divided into two groups on the basis of etiology; the post-tuberculosis bronchiectasis group (Group 1) and the bronchiectasis due to other etiologies nontuberculosis bronchiectasis group (Group 2). Table 1 shows the comparisons of baseline characteristics between Group 1 and Group 2.

**Table 1:** Comparisons of baseline parameters between Group 1 and Group 2

Parameters	Group 1 (n=66)		Group 2 (n=66)		p-Value
	mean	±SD	mean	±SD	
Age	36.08	13.08	46.5	14.17	0.005*
Duration of illness(years)	10.12	8.97	12.16	10.12	0.222
	N	%	N	%	
Gender					
Male	36	54.55	25	37.88	0.48
Female	30	45.45	41	62.12	
Cough	64	48.5	55	41.7	0.019*
Dyspnea	50	37.9	41	31.1	0.132
Chest pain	23	17.4	20	15.2	0.71
Recurrent common cold	17	12.9	54	40.9	<0.001*
Fever	20	15.2	15	11.4	0.43
Haemoptysis	28	42.42	20	30.3	0.205
Smoking	18	27.27	8	12.12	0.049*
Expectoration					
No	4	6.06	7	10.61	0.441
Mucoid	29	43.94	22	33.33	
Mucopurulent	23	34.85	29	43.94	
Purulent	10	15.15	8	12.12	

**Figure 6:**
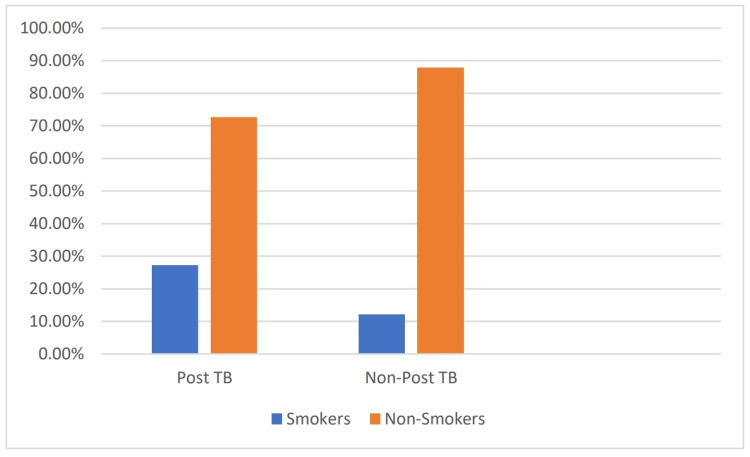
Smoking profile in post-TB vs. non-post-TB bronchiectasis patients

The mean age of patients was significantly lower in Group 1 than in Group 2 (36.08±13.08 vs. 46.5±14.17, p=0.005). The frequencies were significantly higher in patients with smoking and cough and markedly lower in recurrent cold atopy. At the same time, the frequencies of dyspnea, chest pain, fever, and expectoration were not significantly different between groups. The number of smokers (27.27%) was more prevalent in the post-tuberculosis bronchiectasis group (Figure 6).

Hemoptysis was more commonly (42.42%) seen in Group 1 but is nonsignificant. The most common symptoms were cough (48.5%), followed by hemoptysis (42.42%), dyspnea (37.9%), chest pain (17.4%), and fever (15.2%) in post-TB bronchiectasis patients. Cough was significantly higher in Group 1 (48.5% vs. 41.7%, p=0.019). Mucoid expectoration was common in group one; however, mucopurulent expectoration was common in Group 2 but the difference was non-significant (43.94% vs. 43.94%, p=0.44).

Table 2 shows comparisons of general examination, radiological findings, and microbiology between the two groups.

**Table 2:** Comparisons of general examination, radiological findings and microbiology between the two groups

	Group 1 (n=66)		Group 2 (n=66)		p-Value
	n	%	n	%	
Examination					
Anaemia	31	46.97	27	40.91	0.899
Clubbing	43	65.15	43	65.15	1.00
Crepitus	63	95.45	57	86.36	0.130
Rhonchi	38	57.58	32	48.48	0.383
Radiological finding					
Cylindrical	31	46.97	20	30.30	0.074
Varicose	12	18.18	11	16.67	0.819
Cystic	44	66.67	59	86.36	0.014^*^
Tractional	26	39.39	15	22.73	0.060
Centrally located	50	75.76	52	78.79	0.836
Peripherally located	44	66.67	38	57.58	0.370
RUL	27	40.91	33	50.00	0.382
RML	32	48.48	35	53.03	0.728
RLL	31	46.97	38	57.58	0.296
LUL	29	43.94	32	48.48	0.727
LINGULA	36	54.55	35	53.03	0.861
LLL	38	57.58	43	65.15	0.475
Chest CT Sign					
Tram track	49	74.24	28	42.42	<0.001^*^
Signet ring	51	77.27	58	87.88	0.169
Finger in gloves appearance	03	4.55	14	21.21	0.009^*^
Fibrocavitatory changes	10	15.15	02	3.03	0.034^*^
Consolidation	03	4.55	06	9.09	0.490
Microbiology					
Pseudomonas Aeruginosa	13	19.4	10	15.6	-
Staphylococcus aureus	09	13.9	05	7.6	-
Hemophilus influenza	03	4.2	05	7.6	-
Enterococcus sp.	01	1.4	02	3.0	-
Acinetobacter	4.0	6.9	01	1.5	-
Proteus	01	1.5	02	2.8	-
Streptococcus	02	2.8	01	1.5	-

The frequencies of physical examination such as anaemia, clubbing, crepitus, and rhonchi were not significantly different between the two groups. Anaemia was present in 46.97% of patients in the post-tuberculosis bronchiectasis group. Clubbing was seen in 65% of the post-tuberculosis bronchiectasis group. The most common auscultatory finding was crepitations seen in 95.45% of Group 1. Cystic bronchiectasis was the dominant pattern in HRCT in non-tuberculosis bronchiectasis. The difference was significant (86.36% vs. 66.67%, p=0.014); the proportion of tractional and cylindrical bronchiectasis was higher in the post-TB bronchiectasis group. The "tram track" appearance and fibro-cavitary changes were significantly higher in the post-tuberculosis bronchiectasis group. The "finger-in-glove" appearance and "signet ring" sign were considerably lower in Group 1, and the difference was significant. The distribution of bronchiectasis according to the lobes affected showed almost equal distribution in both groups: the most common site of infection was the left lower lobe followed by the lingula, right middle lobe, right lower lobe, left upper lobe, and right lower lobe. The mean serum IgE was lower in post-TB bronchiectasis but not significant (763.57±1624.14 vs. 1038.1±3148.56, p=0.589). Overall, serum IgE levels were elevated in 86.4% study population. Absolute eosinophil counts were also lower in the post-tubercular group (303.45±273.68 vs. 343.00±261.41, p=0.4). Both groups showed no significant differences in serum IgE and absolute eosinophil count (AEC) levels.

On sputum culture, Pseudomonas aeruginosa infection (19.4%) was followed by Staphylococcus aureus (13.4%), most commonly seen in both groups. P. aeruginosa was the most often isolated pathogen in 13 (19.4%) of the post-tubercular patients, followed by Staphylococcus aureus (9; 13.9%), Acinetobacter sp., (4; 6.9%), Hemophilus influenza (3; 4.2%). The comparison of lung function between the two groups showed that mean FEV1 pre, FEV1 post, FVC pre, and FVC post were not significantly different between groups. The FEV1/FVC pre and FEV1/FVC post were significantly lower in Group 1 than in Group 2 (69.16+15.20 vs. 76.86± 15.41, p=0.009 and 70.31±15.56 vs. 76.85 ±11.82, p=0.015) (Table 3).

**Table 3:** Comparisons of lung function test and serum IgE, serum AEC in-between Group 1 and Group 2

	Group 1 (n=66)		Group 2 (n=66)		p-Value
	Mean	±SD	Mean	±SD	
FEV1 pre	1.48	0.53	1.57	0.66	0.457
FEV1 post	3.98	17.26	1.71	0.66	0.327
FVC pre	2.07	0.62	2.00	0.68	0.585
FVC post	3.16	6.81	2.21	0.72	0.301
FEV1/FVC pre	69.16	15.20	76.86	15.41	0.009^*^
FEV1/FVC post	70.31	15.56	76.85	11.82	0.015^*^
S.AEC	303.45	273.68	343.00	261.41	0.490
S.IGE	763.57	1624.14	1038.1	3148.56	0.589

The binary logistic regression was used to find the independent risk factor for post-TB bronchiectasis only; recurrent cough and cold were considered significant independent risk factors for post-TB bronchiectasis. Other variables included age, sex, smoking, FEV1 pre, FEV1 post, FVC pre, FVC post, FEV1/FVC pre, FEV1/FVC post, AEC, serum IgE, duration of illness, and pseudomonas in culture were considered not significant and independent of risk factors for post-TB bronchiectasis (Table 4).

**Table 4:** Binary multivariate logistic regression (forward‑Wald) analysis performed in each patient (diseased) group to determine the independent risk factors for post-TB bronchiectasis

Risk factors	B	Std. Error	Wald	p-Value	Exp (B)	Lower Bound	Upper Bound
Age	0.005	0.031	0.022	0.882	1.005	0.946	1.067
Sex	0.515	0.739	0.487	0.485	1.674	0.394	7.122
Smoking	-0.527	1.148	0.21	0.646	0.591	0.062	5.607
Recurrent cough cold	-2.14	0.93	5.294	0.021^*^	0.118	0.019	0.728
FEV1 pre	1.134	2.039	0.309	0.578	3.107	0.057	169.182
FEV1 post	-0.287	1.691	0.029	0.865	0.75	0.027	20.654
FVC pre	0.725	1.494	0.235	0.628	2.065	0.11	38.617
FVC post	-1.088	1.329	0.67	0.413	0.337	0.025	4.562
FEV1/FVC pre	-0.031	0.06	0.26	0.61	0.97	0.862	1.091
FEV1/FVC post	0.027	0.062	0.184	0.668	1.027	0.91	1.159
AEC	0.001	0.002	0.142	0.706	1.001	0.998	1.004
Serum IgE	0.254	0.732	0.121	0.728	1.289	0.307	5.408
Duration of illness	-0.149	0.058	6.574	0.01	0.862	0.769	0.965
Pseudomonas	-0.326	0.715	0.207	0.649	0.722	0.178	2.933

## Discussion

India has a high population and a high prevalence of tuberculosis. Laennec first noted the co-existence of bronchiectasis and TB in 1819. The following pathogenesis theory of bronchiectasis states that bronchiectasis occurs because of the destruction of the elastic and muscular components of the bronchial wall, usually due to acute or chronic infection. Traction bronchiectasis is a common finding in post-TB sequelae patients. It occurs due to the destruction and fibrosis of the lung parenchyma with irreversible secondary bronchial dilatation [10,11]. 

The study showed that India's most common etiology of non-cystic fibrosis bronchiectasis was post-tubercular, unlike Europe and the USA. The prevalence of tuberculosis is very high in India; as a result, the most common etiology of bronchiectasis is tuberculosis in India [12,13]. As in our study, 50% population had post-tubercular bronchiectasis. A systematic review showed that bronchiectasis was seen in 40-80% of treated cases of tuberculosis [14].

The dominant symptoms in the study group were chronic cough with expectoration and dyspnea, with rates comparable to previously reported series. Haemoptysis, the most devastating sign of bronchiectasis, was noted with a frequency of 42.42% vs 30.30% among patients with post-TB bronchiectasis and non-tuberculous post-infectious, respectively. Post-tubercular bronchiectasis patients had a more significant degree of pulmonary function impairment. Post-tubercular bronchiectasis is an emerging phenotype with high male preponderance, younger age, smokers, and poor lung function. Cough and hemoptysis were the most common presenting symptoms. The most commonly affected age group by bronchiectasis is the 60-70-year age group, and most were females, in line with previous studies [15]. 

Post-tubercular bronchiectasis patients were in the younger age group than 50 years of age. The majority were male. Cough followed by hemoptysis were the most frequent symptoms with daily expectoration in post-tubercular bronchiectasis. Mucoid expectoration was seen in the post-tubercular bronchiectasis group, comparable with the EMBARK study from India [5]. Interestingly, despite numerous patients having post-tubercular bronchiectasis, they complained of day-by-day sputum generation rather than dry bronchiectasis. The productive cough in this study can be clarified by the truth that post-TB sequelae are not limited to bronchiectasis. These patients can have bronchial mutilation, obstructive airway malady disconnected to bronchiectasis, fibrosis, cavitation, etc. [16]. For the same reasons, despite having localized bronchiectasis, some patients had a severe hindrance or nonspecific impairment with exceptionally low FEV1 and FVC. Thus in post-TB patients, it is difficult to comment on the chance that all side effects are due to bronchiectasis only. Among the etiologies, post-infectious causes prevail in this cohort, with 50% of patients having a history of pulmonary TB and 36.36% of patients having a history of other infections in the past.

The prevalence of post-TB bronchiectasis is steady with the high burden of TB in South Asia, with India being positioned second among countries with a high TB burden according to the WHO TB report of 2021. Our study found that the extent of post-TB bronchiectasis is even greater than the data in the information from the previously published Indian bronchiectasis registry, i.e., 50% vs. 35.5% [5]. This is probably the case as our institute established this registry as one of India's biggest nodal TB centers. In our study, 4.54% of the cases stay idiopathic, similar to the more significant part of the past studies where idiopathic cases contain 18-55% of the study population [17].

About 70% were nonsmokers; however, the proportion of smokers was higher in the post-TB bronchiectasis group. Although smoking is not directly associated with bronchiectasis, it causes a worse outcome. In our study, pulmonary function showed obstructive (55%), restrictive (25%), and mixed patterns (15%). Previous studies also showed obstructive impairment was the predominant spirometric pattern [18]. Patients with post-tubercular bronchiectasis had a greater degree of impedance than other types. Pseudomonas was the major ordinary pathogen confined in microbiology, which is consistent with recent data; however, it varies from other studies where Haemophilus influenza was the most standard organism among bronchiectasis patients. In previous studies, Pseudomonas colonization was the critical factor in more severe outcomes among bronchiectasis patients. 

Mean serum IgE and absolute eosinophil counts were lower in the post-tubercular group. Serum IgE and eosinophil counts were performed in all patients to know the etiology of bronchiectasis. However, overall serum IgE was raised in 86.4% study population. The Korean Multicentre Bronchiectasis Audit and Research Collaboration (KMBARC) showed that 19.7% had post-tubercular bronchiectasis and a more severe radiological extent. In this registry 65.7% post-TB bronchiectasis group had obstructive airway disease and lower FEV1 than other groups [19]. Our study also showed that 55% post-tuberculosis bronchiectasis group had obstructive airway disease followed by restrictive and mixed patterns in 25% and 15%, respectively. Some of the patients (5%) could not perform spirometry. Patients with post-tubercular bronchiectasis were predominantly males, younger, and had lower lung function as well as more significant functional impairments. Cough and hemoptysis were the most common presenting symptoms.

## Conclusions

According to our research, post-TB bronchiectasis affected around half of the individuals with bronchiectasis. The individuals who had post-TB bronchiectasis were younger, coughed more frequently, had colds, and smoked more. In post-TB bronchiectasis, fibrocavitatory changes, cylindrical and varicose types of bronchiectasis, and the tram track sign on the CT thorax were frequently observed. In bronchiectasis groups, there were no appreciable intergroup differences in the site of involvement. Patients who had post-TB bronchiectasis had worse lung function than those who had the other types of bronchiectasis.

## References

[1] Hill AT, Sullivan AL, Chalmers JD (2019). British Thoracic Society Guideline for bronchiectasis in adults. Thorax.

[2] Ringshausen FC, de Roux A, Pletz MW, Hämäläinen N, Welte T, Rademacher J (2013). Bronchiectasis-associated hospitalizations in Germany, 2005-2011: a population-based study of disease burden and trends. PLoS One.

[3] Quint JK, Millett ER, Joshi M (2016). Changes in the incidence, prevalence and mortality of bronchiectasis in the UK from 2004 to 2013: a population-based cohort study. Eur Respir J.

[4] Chandrasekaran R, Mac Aogáin M, Chalmers JD, Elborn SJ, Chotirmall SH (2018). Geographic variation in the aetiology, epidemiology and microbiology of bronchiectasis. BMC Pulm Med.

[5] Dhar R, Singh S, Talwar D (2019). Bronchiectasis in India: results from the European Multicentre Bronchiectasis Audit and Research Collaboration (EMBARC) and Respiratory Research Network of India Registry. Lancet Glob Health.

[6] Araújo D, Shteinberg M, Aliberti S (2018). The independent contribution of Pseudomonas aeruginosa infection to long-term clinical outcomes in bronchiectasis. Eur Respir J.

[7] Meghji J, Nadeau G, Davis KJ, Wang D, Nyirenda MJ, Gordon SB, Mortimer K (2016). Noncommunicable lung disease in Sub-Saharan Africa. A community-based cross-sectional study of adults in urban Malawi. Am J Respir Crit Care Med.

[8] Hall GL, Stanojevic S (2019). The Global Lung Function Initiative (GLI) Network ERS Clinical Research Collaboration: how international collaboration can shape clinical practice. Eur Respir J.

[9] Patel AR, Patel AR, Singh S, Singh S, Khawaja I (2019). Treating allergic bronchopulmonary aspergillosis: a review. Cureus.

[10] Bhatta N, Dhakal SS, Rizal S, Kralingen KW, Niessen L (2008). Clinical spectrum of patients presenting with bronchiectasis in Nepal: evidence of linkage between tuberculosis, tobacco smoking and toxic exposure to biomass smoke. Kathmandu Univ Med J (KUMJ).

[11] Jabeen K (2016). Pulmonary infections after tuberculosis. Int J Mycobacteriol.

[12] Leung AN (1999). Pulmonary tuberculosis: the essentials. Radiology.

[13] Aksamit TR, O'Donnell AE, Barker A (2017). Adult patients with bronchiectasis: a first look at the US bronchiectasis research registry. Chest.

[14] Ravimohan S, Kornfeld H, Weissman D, Bisson GP (2018). Tuberculosis and lung damage: from epidemiology to pathophysiology. Eur Respir Rev.

[15] Dimakou K, Triantafillidou C, Toumbis M, Tsikritsaki K, Malagari K, Bakakos P (2016). Non CF-bronchiectasis: Aetiologic approach, clinical, radiological, microbiological and functional profile in 277 patients. Respir Med.

[16] Polverino E, Goeminne PC, McDonnell MJ (2017). European Respiratory Society guidelines for the management of adult bronchiectasis. Eur Respir J.

[17] Angrill J, Agustí C, de Celis R (2002). Bacterial colonisation in patients with bronchiectasis: microbiological pattern and risk factors. Thorax.

[18] Xu JF, Ji XB, Li HB (2013). Bronchiectasis caused by pulmonary tuberculosis: The epidemiology, clinical presentations and the differences from non-tuberculosis-caused bronchiectasis. Eur Respir J.

[19] Choi H, Lee H, Ra SW (2021). Clinical characteristics of patients with post-tuberculosis bronchiectasis: findings from the KMBARC registry. J Clin Med.

